# The Relationship between Infant Colic and Migraine as well as Tension-Type Headache: A Meta-Analysis

**DOI:** 10.1155/2019/8307982

**Published:** 2019-06-16

**Authors:** Dongli Zhang, Yuan Zhang, Yan Sang, Nuo Zheng, Xiaoming Liu

**Affiliations:** Second Ward of Department of Neurology, XuZhou Children's Hospital, Xuzhou Medical University, Xuzhou, Jiangsu, China

## Abstract

**Background:**

Infant colic is a common benign disease during early infancy. Migraine and tension-type headache (TTH) are the most common primary headache forms among pediatric population. Several studies have investigated the incidence of infant colic in patients with migraine and TTH. The meta-analysis was to assess the relationship between infant colic and migraine as well as TTH.

**Methods:**

PubMed, Web of Science, and Cochrane Library were searched until August 16, 2018, for potential studies. Data were extracted by two independent authors and analyzed using RevMan 5.2 software. Odds ratios (ORs) with 95% confidence intervals (CIs) were calculated to determine the association between infant colic and migraine as well as TTH, respectively.

**Results:**

A total of 148 studies were found, and 7 studies were finally included. A higher incidence of colic during infancy was revealed in migraine patients than controls (*P*=0.05, OR: 2.51, 95% CI: 1.32–4.77) and TTH subjects (*P*=0.02, OR: 0.33, 95% CI: 0.13–0.86), respectively. And no significances were found between TTHs with controls (*P*=0.51, OR: 1.17, 95% CI: 0.73–1.89).

**Conclusion:**

This meta-analysis indicated that migraine was associated with increased incidence of infantile colic history, but TTH incidence was not relevant with the incidence of infantile colic history.

## 1. Introduction

Infantile colic is a benign disease during the first months of life and is also one of the most distressing challenges for parents [1]. It is estimated that it affects 10% to 40% of the normal infant population in the world. An infant with this disorder has paroxysms of inconsolable crying for more than 3 hours every day, more than 3 days every week, and longer than 3 weeks [2]. Boys and girls may have equal chances to develop infant colic, and no correlation has been identified with respect to feeding methods, gestational age, and socioeconomic conditions of their families [3]. The pathogenesis of this disease has not been well established [4]. Fecal microflora, gastrointestinal immaturity, and serotonin activity may be responsible for it [2, 5]. It is believed that these infants are sensitive to the environment, and they may present crying to the stimuli.

Headache is a symptom in children and adolescents that usually works as cause for a pediatric clinic consultation [6]. Migraine and tension-type headache (TTH) are the most common primary headache forms among pediatric population, although other primary headache disorders could also be encountered, including cluster headache, paroxysmal hemicranias, and trigeminal autonomic cephalalgia [7]. Migraine is a highly prevalent cause of disability worldwide. It is characterized by recurrent episodes of headache attacks with symptoms, including visual changes and other performances [8]. TTH is also a common disabling condition, with a wide prevalence range from 1.3% to 65% in men and 2.7% to 86% in women [9]. It presents a form of frequent mild to moderate headache, but not associated with migraine symptoms of nausea, vomiting, photophobia, and phonophobia.

A child with headache symptoms also presents a sensitive state to external changes around him or her, like one with infant colic. Then, it is speculated whether the two have some connections. Several studies have investigated the association between infantile colic and migraine as well as TTH [10, 11]. Patients with migraine are likely to have a medical history of infant colic, and an infant with colic tends to have parents with migraine [12, 13]. And infant colic is considered to be early expression of migraine [14]. TTH and migraine exhibit similar pathogenic mechanisms, resulting from the dysfunction of nociceptive pain processing [15]. Then, TTH may also be associated with infant colic. But few studies could be found assessing that between infant colic and TTH. Here, we aimed to investigate the association between infantile colic and migraine, and the relationship between infantile colic and TTH was also studied.

## 2. Materials and Methods

### 2.1. Search Strategy

The meta-analysis was done according to the Preferred Reporting Items for Systematic Reviews and Meta-Analyses (PRISMA) [16]. As this was a meta-analysis, no patient consent was needed for this study. A systematic literature search was done in PubMed, Web of Science, Cochrane Library, and EMBASE until April 5, 2018, to detect the studies assessing the relationship between infantile colic and migraine as well as TTH. The search terms used to retrieve relevant publications included the following in various combinations: “pediatric OR child OR children OR infant OR infantile” and “migraine OR headache OR tension-type headache.” No publication language and study-type restriction were put on this process.

### 2.2. Inclusion Criteria

Studies fulfilling the following requirements were included: (1) a diagnosis of colic within 3 months old, (2) clinical articles, and (3) full record of the incidence of infant colic in migraine or detailed information could be retrieved through contacting the authors. The diagnosis of migraine and TTH were defined according to the criteria of the International Classification of Headache Disorders III; (ICHD III;) [17]. Wessel criteria [18] were used for infant colic as more than 3 hours per day crying, more than 3 days a week, and more than 3 weeks during infancy. Details of diagnostic characteristics of migraine, TTH, and infant colic could be found in Supplementary Table 1.

### 2.3. Data Collection

Data were manually extracted from each report by two independent authors, and any disagreement was determined by the senior author (the corresponding author). Data were collected, including authors, publication year, publication type, study period, age, no. of patients and controls, and incidence of infant colic in control, migraine, and TTH.

### 2.4. Quality Assessment

The quality of included studies was analyzed using the Newcastle–Ottawa quality assessment scale (NOS) [19], including representativeness of cases, selection of the nonexposed cases, ascertainment of exposure, demonstration of not presenting the outcome of interest at the start of the study, comparability of the analysis, assessment of outcomes, duration of follow-up, and lost to follow-up. The item with highest quality in NOS was awarded with a maximum of one star with the exception of the item related to comparability rated with two stars. Then, the total score of each study ranged between 0 and 9 stars. Studies with 6 stars or more were considered with relatively high quality.

### 2.5. Statistical Analysis

Statistical analysis was done using RevMan 5.3 software package (the Cochrane Collaboration, Copenhagen, Denmark). Dichotomous outcomes were expressed as odds ratios (ORs) with 95% confidence intervals (CIs). Heterogeneity among studies was analyzed using *I* ^2^ test methods, with *I* ^2 ^≥^ ^50% or *P* < 0.05 as an indicator of a high level of heterogeneity. Fixed effects (*I* ^2^ < 50%) and random effects (*I* ^2^ ≥ 50%) models were selected according to the level of heterogeneity among the trials. The significance level was set at *P* < 0.05. Publication bias was detected when there were more than 10 studies in the comparisons, by observing the funnel plots.

## 3. Results

### 3.1. Search Results and Study Characteristics

**Figure 1:**
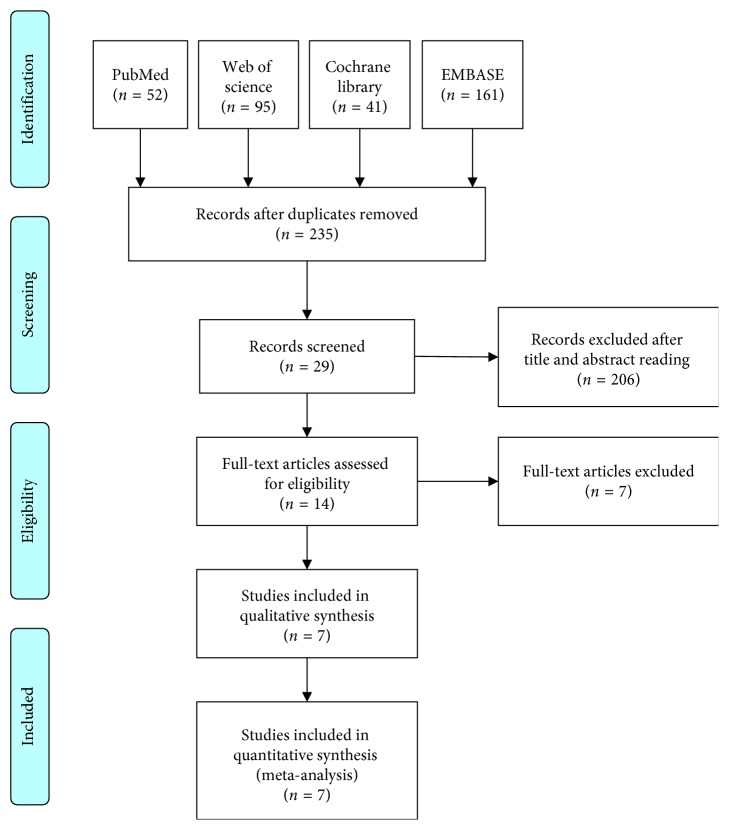
The flow diagram of the meta-analysis.

The flow diagram of the study is shown in Figure 1. The initial search retrieved 349 records from PubMed, Web of Science, Cochrane Library, and EMBASE. Then, 114 duplicates were removed. Further assessment of titles and abstracts identified 29 papers. Full-text assessment further excluded 7 articles. The final inclusion then confirmed 7 articles in the study [10–12, 20–23]. The baseline characteristics of them are displayed in Table 1. They were published between 1997 and 2017, involving 7 countries: Italy, Saudi Arabia, USA, France, Spain, Finland, and Iran. One study was an international study based on people in France and Italy [10]. All of them were published in English language. Two studies included adult patients [21, 22], and 5 trials enrolled pediatric population [10–12, 20, 23]. Only one study did not indicate age of the included studies [11]. The diagnosis of infant colic was made based on medical history provided by parents in 4 trials [10, 11, 20, 23], two studies confirmed infant colic through follow-up visiting and diagnosis by physicians [22, 24], and one study got the record from examinee questionnaire [21]. All the studies compared the occurrence of colic in migraine and control groups, and 2 trials of them also recorded that in TTH patients [10, 11]. A total of 3174 subjects were enrolled, of which 606 were migraine sufferers, 239 were TTH patients, and 2329 were controls. The results of quality assessment are also shown in Table 1.

### 3.2. Outcomes

**Figure 2:**
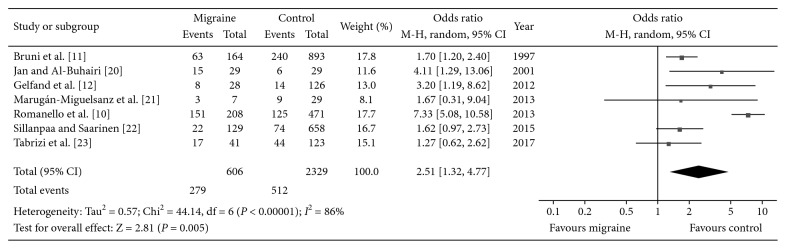
Forest plot of the incidence of infant colic between migraineurs and controls.

**Figure 3:**
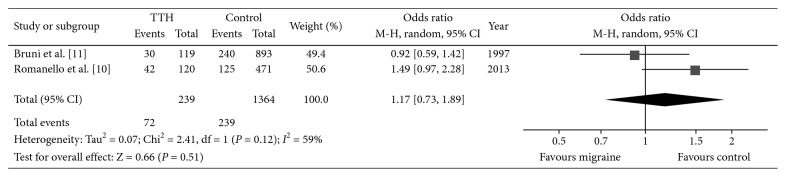
Forest plot of the incidence of infant colic between TTHs and controls. TTH, tension-type headache.

**Figure 4:**
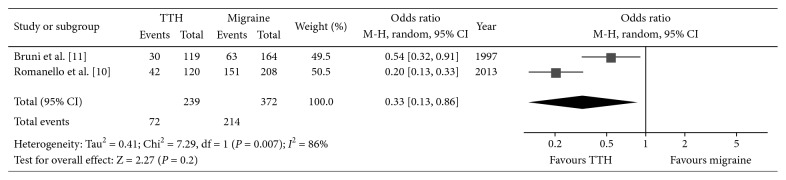
Forest plot of the incidence of infant colic between migraineurs and TTHs. TTH, tension-type headache.

The outcomes in this study are depicted in Table 2. A higher incidence of colic during infancy was found in migraine patients than controls (*P*=0.005, OR: 2.51, 95% CI: 1.32–4.77), with high heterogeneity (*I* ^2^ = 86%; Figure 2). However, no significance was found when comparing TTH patients with controls (*P*=0.51, *I* ^2^ = 59%, OR: 1.17, 95% CI: 0.73–1.89; Figure 3). We also analyzed the infantile colic incidence between migraine and TTH subjects, and a higher incidence was also seen in migraineurs (*P*=0.02, *I* ^2^ = 86%, OR: 0.33, 95% CI: 0.13–0.86; Figure 4).

When analyzing data from pediatric population, the results and heterogeneity did not change the trends (*P*=0.01, *I* ^2^ = 90%, OR: 2.89, 95% CI: 1.28–6.50; Table 3). But a mere significance existed in adult population (*P*=0.05, OR: 1.63, 95% CI: 0.99–2.67), with a marked change of heterogeneity from 86% to 0%. Also, since the diagnosis of infantile colic was quite different among the studies, we assessed the pooling data based on them. Medical history was derived from parents in 4 studies, and the trend of results did not change (*P* < 0.0001, *I* ^2^ = 92%; Table 3). Infant colic was defined during follow-up visits in 2 studies, and the pooling data also showed the same trend with the overall outcome with a robust change of heterogeneity (*I* ^2^ = 29%). One study with medical history from patients indicated comparable results between migraine and control groups (*P*=0.55).

### 3.3. Publication Bias Analysis

Since there were less than 10 studies in the comparisons, an accurate publication bias analysis cannot be performed.

## 4. Discussion

This meta-analysis evaluated the association between infantile colic and migraine as well as TTH. It indicated that migraine was associated with increased incidence of infantile colic history. But TTH was not relevant to colic incidence during infancy.

Headache is a widely suffering symptom in children and adolescent which leads to pediatric consultations in the hospital [25]. There are many types of headache among the population. Migraine and TTH are more commonly encountered, and other primary headache disorders are quite rare to be seen [7, 26]. Migraine is a common and chronic disorder with multilevel factors in the neurovascular structure accounting for its recurrent headaches. It comes along with TTH, as the most common paroxysmal diseases during childhood. Although the two have similar headache characteristics in some aspects, they have different performances in neuroimaging studies and have various responses to treatment strategies.

Infant colic was first described in 1954. The term “colic” implies an abdominal etiology with little direct localization evidence. An association between infant colic and migraine has been established in several studies [27–29]. Our study assessed the associations between infant colic and migraine as well as TTH. And we did not find any studies collecting information on other forms of headache. All the studies were with quite high quality, ensuring the credibility of the results. Infant colic may have common pathological and physiological changes with these two headache syndromes.

Seven studies indicated a higher incidence of infant colic in migraine patients, which is consistent with the previous trials. Bruni et al. [11] and Jan and Al-Buhairi [20] found a significant higher colic during infancy in migraineurs. But a high heterogeneity was found in the outcome. We tried to find the source of it. It seemed that age may be a source of the heterogeneity. There was a robust change of heterogeneity in the respective analysis of adult and pediatric subjects. Although similarities do exist in the symptoms and treatment for migraine between adults and children, some differences still can be found between them. Psychological treatment is more effective for children than adults, indicating the high proportion of psychological factors in children migraine [30]. Also the different ways to define the diagnosis of infant colic were also responsible for it. Wessel criteria [18] were used to define the disease. However, only subjects in two studies received the diagnosis through periodic follow-up visiting with the doctors. Other studies collected the history of infant colic through questionnaire or medical history from the parents or the patients themselves. Apparently, the process was done based on the memories of the examinees. This might not be so reliable for long-term memory. The respective analysis of groups based on the ways to make the diagnosis indicated the similar results in 4 studies with medical history from parents and 2 studies from follow-up visiting. But the high heterogeneity disappeared in the latter comparison. Also, no significances were found in the comparison based on medical history from patients. A correlation between colic and sleep disorders has been suggested. Infants with colic usually exhibit their crying and other behaviors during sleep. Migraine patients also have a bad sleep status [31]. Children with migraine are more likely to have colic during infancy, and parents with migraine are more likely to have a baby with colic [12, 20]. It is believed that infants with migraine genetics may be more sensitive to environments than healthy ones [32, 33].

TTH is also a common type of headache among children [6]. But the incidences of infant colic were quite comparable between patients and controls, which indicated no link between the two diseases and the different etiology from migraine. But only two studies investigated the records, encouraging further efforts on these subjects.

There is still no consensus concerning the treatment of infant colic. Increasing attention has been paid to the effects of dietary, pharmacological drugs, and behavioral strategies. Breastfeeding and bottle-feeding infants may have distinguished dietary options: (1) a monitored low allergen maternal diet without cow milk, containing vitamins and minerals, may be appropriate for the former ones and (2) a formula based on casein hydrolysate is recommended for the latter infants. Although simethicone is suggested for infants for its effects on reducing gas production, limited evidences can be provided. The application of probiotics is supported by the idea that the medication can alleviate gut dysfunction caused by abnormal intestinal microflora, then reducing gas production and colic symptoms. Many other therapeutic approaches targeting infant colic can be found from literature search. But the lack of high quality and objective outcome assessing method makes it difficult to get a conclusive guideline for this disorder.

### 4.1. Study Quality

There were also some limitations in this study. First, the number of the included studies was quite limited, limiting the evidence level of this meta-analysis. Second, the definitions of colic in infancy in each study were not so consistent. This might cause the high heterogeneity of the pooling results. Also, no potential mechanisms of infant colic in migraine were assessed in these studies. Further investigation on this field should be done.

## 5. Conclusion

In conclusion, migraine was associated with increased incidence of infantile colic history. TTH was not relevant to colic incidence during infancy. However, large trials, containing more participants, should be included in the future.

## Figures and Tables

**Table 1:** The baseline characteristics of the included studies.

Study	Bruni et al. [11]	Jan and Al-Buhairi [20]	Gelfand et al. [12]	Romanello et al. [10]	Marugán-Miguelsanz et al. [21]	Sillanpaa and Saarinen [22]	Tabrizi et al. [23]
Age	NA	7–12 y	2–12 w	6–18 y	18–45 y	18 y	5–15 y
Type	Prospective	Prospective	Retrospective	Prospective	Retrospective	RCT	Retrospective
Period	1994.09–1995.09	1998.08–1998.12	2010.07–2011.09	2012.04–2012.06	NA	NA	2015–2016
Region	Italy	Saudi Arabia	USA	France and Italy	Spain	Finland	Iran
No.	NA	NA	NA	NA	NA	NA	NA
Con.	893	29	126	471	29	658	123
Migraine	164	29	28	208	7	129	41
TTH	119	NA	NA	120	NA	NA	NA
Colic	NA	NA	NA	NA	NA	NA	NA
Con.	240	6	14	125	9	74	44
Migraine	63	15	8	151	3	22	17
TTH	30	NA	NA	42	NA	NA	NA
Diagnosis of infant colic	Medical history from parents	Medical history from parents	Follow-up visiting	Medical history from parents	Medical history from patients	Follow-up visiting	Medical history from patients
Study quality	6	7	8	7	6	8	6

NA: not applicable, y: year, w: week.

**Table 2:** Meta-analysis results of the included studies.

Outcome	No. of studies	*P*	OR [95% CI]	Heterogeneity			
				*χ* ^2^	df	*I* ^2^ (%)	*P*-*Q* test
Colic in migraine	7	0.005	2.51 [1.32, 4.77]	44.14	6	86	<0.00001
Colic in TTH	2	0.51	1.17 [0.73, 1.89]	2.41	1	59	0.12
Migraine vs. TTH	2	0.02	0.33 [0.13, 0.86]	7.29	1	86	0.007

OR: odds ratio.

**Table 3:** Meta-analysis based on age and diagnosis of colic.

Outcome	No. of studies	*P*	OR [95% CI]	Heterogeneity			
				*χ* ^2^	df	*I* ^2^ (%)	*P*-*Q* test
*Age*							
Adult	2	0.05	1.63 [0.99, 2.67]	0	1	0	0.98
Children	5	0.01	2.89 [1.28, 6.50]	38.77	4	90	<0.00001

*Diagnosis of colic*							
Medical history from parents	4	<0.00001	3.15 [2.52, 3.93]	38.77	3	92	<0.00001
Follow-up visting	2	0.03	1.99 [1.08, 3.66]	1.42	1	29	0.23
Medical history from patients	1	0.55	1.67 [0.31, 9.04]	NA	NA	NA	NA

OR: odds ratio and NA: not applicable.

## Data Availability

The data used to support the findings of this study are available from the corresponding author upon request.

## Supplementary Materials

Supplementary Materials Supplementary Table 1: the diagnostic criteria of migraine, tension-type headache, and infant colic. ICHD, the International Classification of Headache Disorders.
