# Supplementary material for: The Relationship between Infant Colic and Migraine as well as Tension-Type Headache: A Meta-Analysis
Source: Pain Res Manag. 2019 Jun 16;2019:8307982. doi: 10.1155/2019/8307982 (PMC6604354; doi:10.1155/2019/8307982)
Supplement: Supplementary Materials — Supplementary Table 1: the diagnostic criteria of migraine, tension-type headache, and infant colic. ICHD, the International Classification of Headache Disorders. [file 8307982.f1.pdf]

Migraine

Migraine with aura

A. At least five attacks fulfilling criteria B-D

B. Headache attacks lasting 4-72 hours (untreated or unsuccessfully treated)2;3

C. Headache has at least two of the following four characteristics:

1. unilateral location

2. pulsating quality

3. moderate or severe pain intensity

4. aggravation by or causing avoidance of routine physical activity (eg, walking or climbing stairs)

D. During headache at least one of the following: 1. nausea and/or vomiting 2. photophobia and phonophobia

E. Not better accounted for by another ICHD-III diagnosis.

Migraine without aura

A. At least two attacks fulfilling criteria B and C

B. Aura consisting of visual, sensory and/or speech/language symptoms, each fully reversible, but no motor, brainstem or retinal symptoms

C. At least two of the following four characteristics:

1. at least one aura symptom spreads gradually over ≥5 minutes, and/or two or more symptoms occur in succession

2. each individual aura symptom lasts 5-60 minutes

3. at least one aura symptom is unilateral

4. the aura is accompanied, or followed within 60 minutes, by headache

D. Not better accounted for by another ICHD-III diagnosis, and transient ischaemic attack has been excluded.

Tension-type headache

A. At least 10 episodes of headache occurring on <1 day per month on average (<12 days per year) and fulfilling criteria B-D

B. Lasting from 30 minutes to 7 days

C. At least two of the following four characteristics:

1. bilateral location

2. pressing or tightening (non-pulsating) quality

3. mild or moderate intensity

4. not aggravated by routine physical activity such as walking or climbing stairs

D. Both of the following:

1. no nausea or vomiting

2. no more than one of photophobia or phonophobia

E. Not better accounted for by another ICHD-III diagnosis.

Infant colic

For clinical purposes, must include all of the following:

1. An infant who is ＜5 months of age when the symptoms start and stop

2. Recurrent and prolonged periods of infant crying, fussing, or irritability reported by caregivers that occur without obvious

cause and cannot be prevented or resolved by caregivers

3. No evidence of infant failure to thrive, fever, or illness

For clinical research purposes, a diagnosis of infant colic must meet the preceding diagnostic criteria and also include both of the following:

1. Caregiver reports infant has cried or fussed for 3 or more hours per day during 3 or more days in 7 days in a telephone or face-to-face screening interview with a researcher or clinician

2. Total 24-hour crying plus fussing in the selected group of infants is confirmed to be 3 hours or more when measured by at least one prospectively kept, 24-hour behavior diary

Supplemental Table 1. The diagnostic criteria of migraine, tension-type headache and infant colic. ICHD, the International Classification of Headache Disorders.
